# Understanding learners’ experiences across three major transitions in undergraduate medical education

**DOI:** 10.1186/s12909-024-05422-1

**Published:** 2024-07-11

**Authors:** Morgan E. Weyant-Cheeseman, Matthew P. Abrams, Nicholas Toselli, Analia Castiglioni

**Affiliations:** 1 Department of Pediatric Emergency Medicine, University of Texas Southwestern, 1935 Medical District Dr, 75235 Dallas, TX USA; 2 Department of Psychiatry, University of California San Diego, San Diego, CA USA; 3 Department of Emergency Medicine, Orlando Health, Orlando, FL USA; 4 University of Central Florida College of Medicine, Orlando, FL USA

**Keywords:** Medical Education, Clerkships, Nominal group technique

## Abstract

**Background:**

Medical students in the United States undergo three significant transitions as they progress from pre-clinical to clinical rotations, to acting interns, and ultimately to first-year resident. However, there is limited understanding of whether the factors and strategies that impact these transitions remain consistent or are unique to each individual transition.

**Methods:**

Qualitative data was collected from three Nominal Group Technique (NGT) sessions held separately for third-year students (M3), fourth-year students (M4), and first-year residents (PGY-1). The participants were asked to share their perceptions on their most recent medical school transition. These responses were independently coded into thematic categories.

**Results:**

The NGT session with M3 students (*n* = 9) identified 67 transition factors and 64 coping strategies. The M4 (*n* = 8) session identified 33 transition factors and 72 strategies to manage their transition. The PGY-1 (*n* = 5) session identified 28 factors and 25 strategies. The coping strategies for each session were categorized into seven themes and the transition factors were assigned to ten thematic categories.

**Conclusion:**

Just as each transition is unique, so too are the number and variety of factors and strategies to manage each transition. Despite these differences, the themes of “Professional socialization” and “Prioritization, efficiency, and delegation” emerged as impactful in all three transitions.

**Supplementary Information:**

The online version contains supplementary material available at 10.1186/s12909-024-05422-1.

## Background

Transitioning is a multifaceted process of developing new behavioral responses to navigate through significant disruptions in an individual’s life space [1]. Traditionally, U.S. medical students undergo three major transitions throughout the course of undergraduate medical education: (1) from pre-clinical years to clinical rotations, (2) from standardized core clerkship rotations to the flexibility of electives combined with the role of acting intern in the fourth year, and (3) from fourth-year medical student to first-year resident or intern [2]. These transitions play a crucial role in shaping medical students’ professional identity formation and personal development [3]. However, these transitions are marked by periods of substantial changes in context and responsibilities, presenting new challenges and heightened stress levels [4–7]. These challenges can have a detrimental impact on learning, as the stages are inherently different [8, 9].

The preclinical years of medical school are distinctly different from the clinical years [10]. In the classroom setting, students acquire knowledge within a structured environment that focuses on objective goals and content, prioritizing the students as learners [9]. In contrast, during clerkships, acting-internships, and residencies, learning takes on an experiential nature, encompassing real-life clinical activities primarily oriented toward patient care [8, 11–14]. When transitioning between these environments, students face various learning challenges, necessitating adjustment to the culture, development of task mastery, clarification of roles, and integration into the social fabric [15].

While multiple studies have investigated the attitudes, challenges, and coping mechanisms facing learners during each individual transition [2, 16], few have examined these processes along the undergraduate and graduate medical education (GME) continuum. Specifically, no studies have investigated and compared the perceptions and management strategies employed by learners during the three major transitions. Therefore, this study aimed to gain a deeper understanding of the cross-sectional experiences of medical students during these three major transitions at the University of Central Florida College of Medicine (UCF COM): (1) from pre-clinical years to clinical rotations (M3), (2) from standardized core clerkship rotations to the flexibility of electives combined with the role of acting-intern in the fourth year (M4), and (3) from fourth-year medical student to first-year resident (intern or PGY-1). Using a mixed-methods, trainee-oriented approach we sought to: (1) identify factors that define students’ transitions, (2) identify the coping strategies utilized during each transition, and (3) compare and contrast transition factors and management strategies in the three major transitions of undergraduate medical education.

## Methods

### Institutional context

The University of Central Florida College of Medicine (UCF COM) provides a four-year M.D. program. The initial two years are focused on pre-clinical education, and interspersed throughout this time, students engage in clinical skills training using standardized patients and gain exposure to clinical settings. The third year consists of core clerkship rotations in set specialties including Internal Medicine, Family Medicine, Pediatrics, Surgery, Obstetrics and Gynecology, Psychiatry, and Neurology, where students gain hands-on experience in diverse clinical settings and start to develop their clinical competence. The fourth and final year of the M.D. program is designed to offer students more flexibility and autonomy, and students can tailor their schedules according to their career interests and goals, encouraging exploration of new specialties. As part of the fourth year, students are required to complete acting internships, also known as sub-internships, which provide opportunities for fourth-year students to assume increasing responsibility as they prepare to enter residency. By participating in acting internships, students learn how to function effectively as integral members of a medical team. For many students, these experiences serve as a defining factor in solidifying their career paths and confirming their chosen specialties.

### Participants

M3, M4, and PGY-1 learners were invited to take part in Nominal Group Technique (NGT) sessions held between February– March 2019. An invitational email was sent from the dean’s office to the entire M3 and M4 class (120 students per class), and eight PGY-1 trainees who had graduated from the UCF COM in May 2018 and were enrolled in UCF-affiliated GME programs. Enrollment was on a first-come, first-serve basis. In alignment with NGT methodology, group size was limited to 8–10 participants to ensure the effective conduction of each session. No participant demographic data was collected. Participation was voluntary; all participants received monetary compensation for their time. Gift cards were made available through research funding provided by an institutional grant from the Department of Internal Medicine at the University of Central Florida. The participants gave their informed consent for inclusion prior to participating in the study and the study was determined exempt from regulation by the University of Central Florida Institutional Review Board (SBE-18-14490).

### Study design

This study utilized a qualitative approach to evaluate the transitions in undergraduate medical education. First, the qualitative phase involveddata collection via Nominal Group Technique (NGT) sessions [17] to gather consensus on the perceptions and experiences that defined each transition. Then, the responses were analyzed and coded into categories. The third part of the study, which will be published later, utilized the top responses from the NGTs to create a survey, which was distributed to subsequent M3, M4, and PGY-1 classes to further evaluate learners’ perceptions of each transition.

#### Nominal group technique sessions

Nominal group technique (NGT) [17, 18] was employed in this study to comprehensively identify and arrive at a consensus about the transition factors and coping strategies that had the greatest influence on the most recent transition experienced by UCF COM students. NGT is a highly structured, multi-step, facilitated group meeting, which is used to elicit and prioritize answers to a single, specific question [17]. The highly structured format of an NGT session promotes equal involvement of all participants, prevents tangential and evaluative discussions often observed in group settings, and minimizes process loss typically associated with unstructured focus groups [18]. By promoting equitable participation and giving equal weight to input from all participants, the data generated through this process is considered a valuable and easily interpretable reflection of the prioritized perspectives held by a representative group.

NGT sessions were conducted in person in a UCF COM conference room, with the same moderators posing the same questions. The moderators were the researchers, both MWC and NT were third-year medical students at UCF COM at the time of the NGT sessions, making them familiar with the curriculum. Separate NGT sessions were held for M3 students, M4 students, and PGY-1s to capture their perceptions regarding their most recent transition in medical education.

Each group was presented with two prompts: *“Think about your transition time from x to y, what strategies did you use to overcome/cope with the transition?”* and *“Think about your transition time from x to y, what factors defined your transition experience?”* Individually, students recorded as many responses as possible to each prompt on paper for a 20-minute timeframe. The participants then shared their answers in a round-robin fashion, with each response being recorded and displayed for all to see. After recording all responses, group discussion established a consensus list and clarified any ambiguities in language. Using the collective responses, participants anonymously assigned points ranging from 1 to 5 to the elements that had the most impact on their respective transitions. The assigned rankings were summed across participants to generate a group-level result. Participants underwent this process first for transition management strategies, then for the factors that defined their transition.

#### Qualitative content analysis

The NGT responses underwent a deductive, qualitative content analysis to code each response into categories. Transition factors were organized according to the transition themes previously identified by Prince et al. [19], whereas the strategies [16] for each transition were categorized based on those identified by the work of O’Dowd and Waqas [2, 20, 21]. Final themes were established through an iterative process and new themes that emerged from the student-generated data were incorporated into the framework. Two members of the team independently assigned categories to each NGT response, then met to discuss and reconcile any differences. A full consensus was reached for each theme. Once the initial coding was completed, a second level of analysis using continuous comparison was undertaken to ensure validity of the categorization. Finally, a third investigator (AC) reviewed, discussed, and validated the coding.

The five themes from Prince et al. [19] were applied to the factors identified by each group, with the modification of two themes to “Knowledge, knowledge application and skills” and “Learning, education, and career development.” Two additional themes were added: “Balance and wellness” and “Non-medical logistics” (e.g. finances, rotation site, roommates). The strategies aligned with eight themes identified by O’Dowd [20], with one modification to “Resilience attitudes and mature coping mechanisms” (e.g. taking one day at a time, motivated self-learning). The inclusion of “Immature coping mechanisms” (e.g. denial, suppressing feelings, trying study drugs) stemmed from the work of Waqas et al [21]., and the final theme, “School/program support and educational tools” was created to capture strategies that relied upon resources provided by the college of medicine or residency program.

Descriptive statistics were performed for the NGT data, including measures of frequency of each theme.

## Results

### Nominal group technique sessions

The M3 NGT session (*n* = 9) identified a total of 67 factors that influenced their transition and 64 strategies to navigate this transition (Appendix 1). By number of points assigned, M3s identified “Lack of autonomy over clerkship hours” and “Maintaining enthusiasm throughout the clerkship” as the most impactful factors of the transition from M2 to M3, whereas the most impactful strategy was “Talking to M4s and PGY-1s about their experiences, recommended resources, and general advice.” Refer to Appendix 1 for the full prioritized, ranked list of factors and strategies generated from the M3 NGT.

In the M4 NGT session (*n* = 8), 33 factors impacting their transition and 72 strategies to manage this transition were identified (Appendix 2). By points assigned, the fourth-year medical students identified “Choosing a specialty” as the most impactful factor, while the most impactful strategy was “Mentorship from alumni and residents.” Refer to Appendix 2 for the full prioritized, ranked list of factors and strategies generated from the M4 NGT.

The PGY-1 session (*n* = 5) identified 28 factors that affected their transition and 25 strategies to cope with the transition (Appendix 3). By points assigned, the first-year residents identified “Number of hours spent at work” as most impactful factor of their transition. Their most impactful strategy was “Vacation time.” Refer to Appendix 3 for the full prioritized, ranked list of factors and strategies generated from the PGY-1 NGT.

**Fig. 1:**
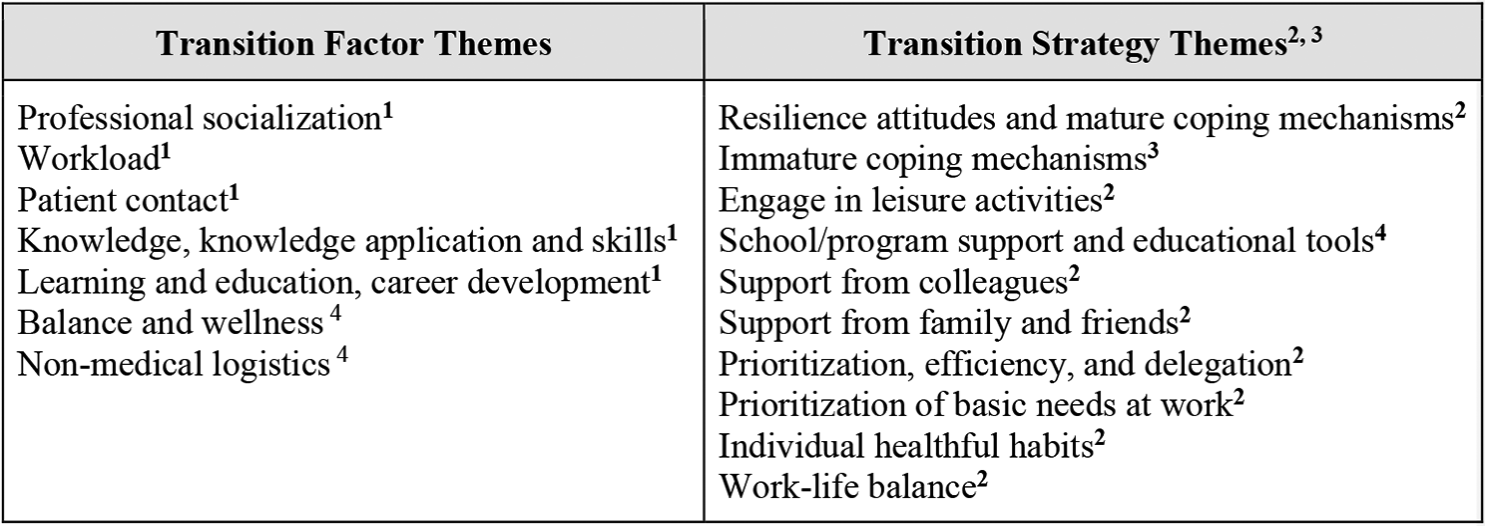
Themes identified during coding with delineation of the source of themes. ^1^ Transition factor themes modified from those identified by Prince et al. 2005. ^2^Transition strategy themes modified from those identified by O’Dowd et al. 2018. ^3^Transition strategy themes modified from those identified by Waqas et al. 2018. ^4^Additional themes not from the cited frameworks

### Qualitative content analysis

**Fig. 2:**
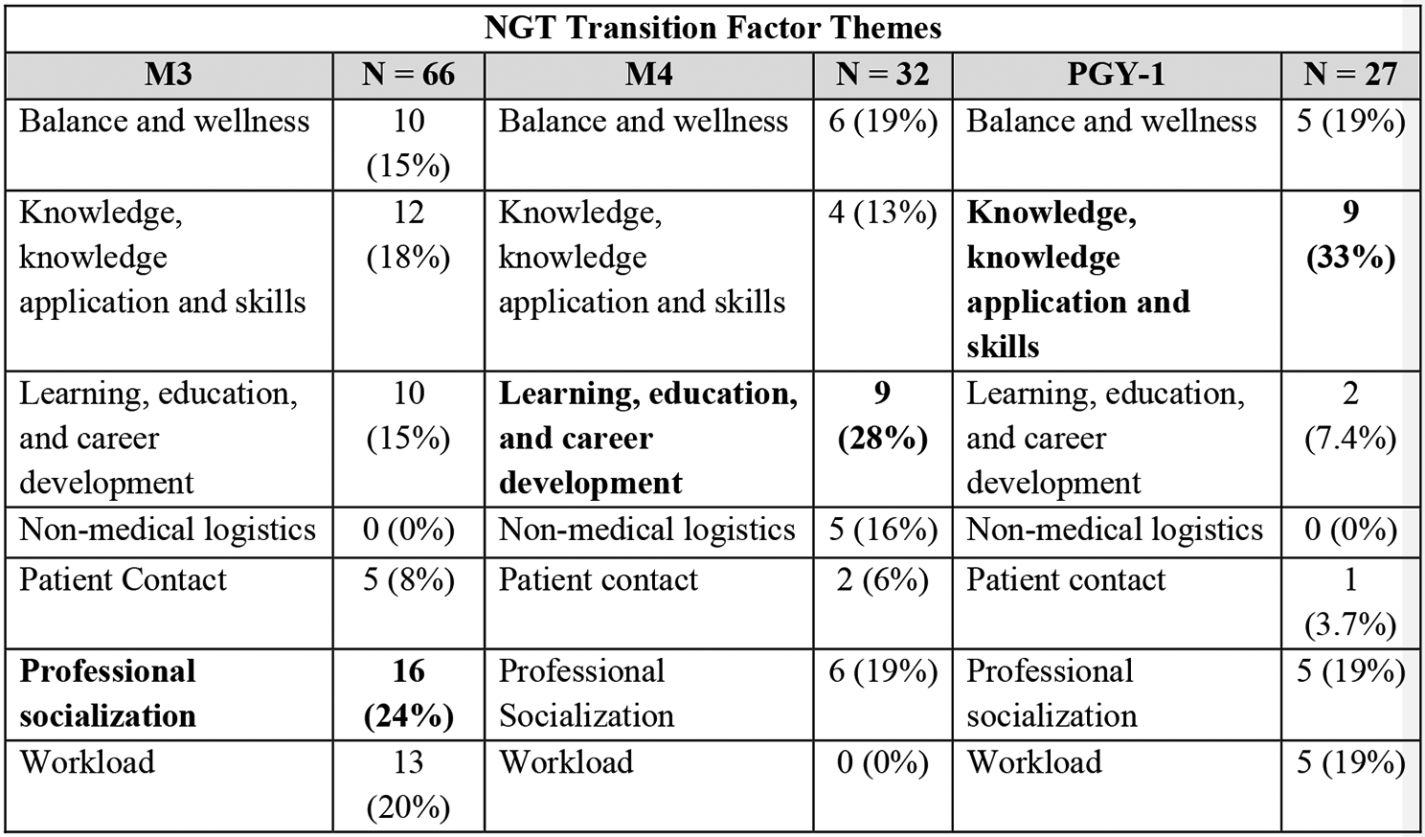
Nominal group technique themes regarding factors that affected the students’ transition and proportion of factors coded to each identified theme. **Bolded** themes indicate the theme with the largest number of factors identified within each group

**Fig. 3:**
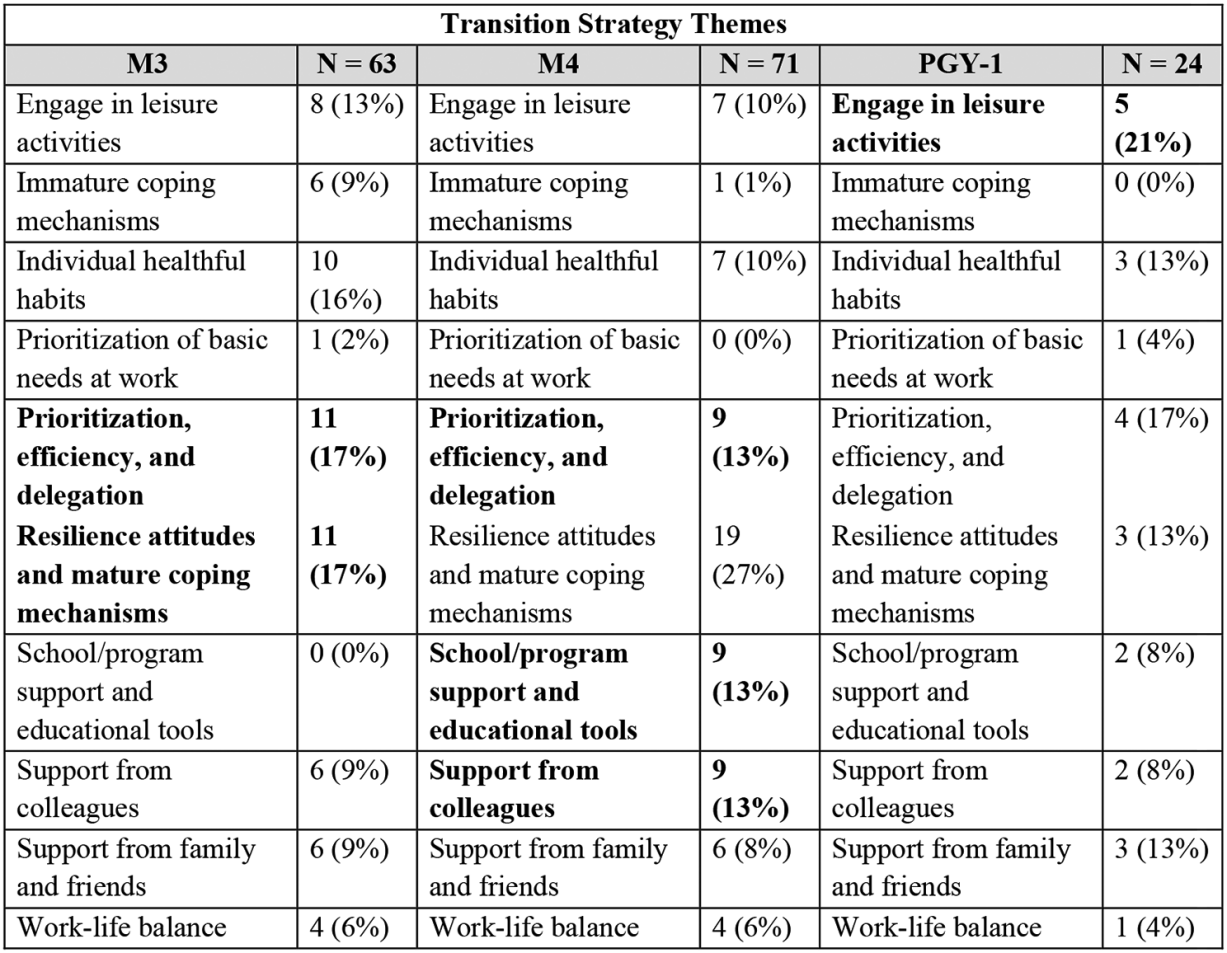
Nominal group technique themes regarding strategies that facilitated the students’ transition and proportion of strategies coded to each identified theme. **Bolded** themes indicate the theme with the largest number of strategies identified within each group

Figure 1 illustrates the final themes, divided into factors and strategies, with further stratification by prevalence in Figs. 2 and 3.

Based on their NGT responses, the M3s most identified factors related to the theme of “Professional socialization” to be impactful to their transition. The most cited transition strategies aligned within the themes of “Prioritization, efficiency, and delegation” and “Resilience attitudes and mature coping mechanisms.” None of the responses generated from the M3 NGT cited factors of “Non-medical logistics” as impactful to their transition or strategies related to the theme of “School/program support and educational tools” to help manage their transition.

In the M4 NGT, the most identified transition factors related to the theme of “Learning, education, and career development.” The fourth-year medical students most cited strategies within the theme of “Resilience attitudes and mature coping mechanisms” to manage their transition. None of the responses generated from the M4 NGT cited “Workload” as a factor impactful to their transition or “Prioritization of basic needs at work” as a strategy to help manage their transition.

The PGY-1 NGT most identified transition factors related to the theme of “Knowledge, knowledge application and skills” to be most impactful. They most cited strategies within the theme of “Engage in leisure activities” to manage their transition. None of the responses generated from the PGY-1 NGT cited “Non-medical logistics” as a factor impactful to their transition or used “Immature coping mechanisms” as a strategy to help manage their transition.

Notably, for all three transitions, students commonly highlighted the themes of “Professional socialization” as impactful to the transition, while each class frequently identified strategies emphasizing “Prioritization, efficiency, and delegation” to help manage the transition. Additionally, while “Workload” was frequently cited as an impactful factor by M3s and PGY-1s, none of the responses generated from the M4 NGT fit into this theme. Conversely, “Non-medical logistics” was only identified as an impactful factor by the M4s. Students cited “Immature coping mechanisms” less frequently as strategies to manage their transitions as they advanced through medical training, with some increase in “Resilience attitudes and mature coping mechanisms.”

## Discussion

This study sought to gain a better understanding of the factors and strategies that are most important during the three major transitions in undergraduate and early graduate medical education. Unique about this study is its cross-sectional nature, in that all three transitions were examined simultaneously. To our knowledge, no studies have previously evaluated and compared the factors that define and coping strategies employed by learners during these three major transitions.

When looking at these three transitions together, our results show that learners perceive multiple factors and strategies as contributing to transitions in medical education. The number and variety of factors and strategies identified by each NGT group speak about the complexity of these junctures, and the variability in individual experiences.

Review of identified themes across all three transitions identifies what they have in common and where they differ. For example, professional socialization consistently emerges as one of the most impactful themes across all transitions, supporting the importance of ongoing professional identity formation throughout the educational continuum. However, when looking at the factors contributing to this theme across transitions, one can observe a negative influence of socialization factors in the M3 transition (e.g. trying to impress attendings and residents, always being watched, navigating different personalities of superior), versus a positive impact of those mentioned by M4s (e.g. no longer most junior member of team, better established relationship with peers and mentors, relationship with attendings and nurses) and PGY-1s (e.g. figuring out new expectations, finding good mentors who understand my career path). The different impact professional socialization factors have on each transition can help guide the timing and type of interventions offered to learners. For example, peer mentoring that is aimed at teaching the hidden curriculum of medicine and the necessary skills for clinical success can be offered at the clerkship level, while faculty mentoring, career planning and teamwork skills can be offered at later transitions. We confirmed that transition strategies such as building resilience, engaging in self-care, and learning to be efficient and prioritize work are also represented in all transitions, which suggests further opportunities for creation or growth of curricular interventions. Furthermore, learners across all transitions also consistently cited the importance of near peers and faculty as important strategies to facilitate their adjustment to each new learning environment, and also found value in disengaging from the stress of training through leisure activities.

Although the transition from the classroom to the clinical setting in the third year of medical school is eagerly anticipated by most students, many struggle with the shift from a student-centric learning environment to one that is focused on patient care [9]. Some students feel unprepared for clinical rotations, experience stress and anxiety, and find it challenging to engage in patient interactions, resulting in decreased performance and hindering learning [2, 4]. Further, adjusting to the increased workload, unclear roles and expectations, and limited study time poses difficulties for many students. Previous research has demonstrated that early patient contact during the preclinical years may decrease the “shock of practice” and ease student transitions by preparing them earlier for clinical interactions [22]. However, our findings indicate that M3 students enjoy patient care and increasing responsibility associated with growing autonomy, supporting the notion that factors other than patient care may contribute to the difficulties of the transition. As mentioned above, professional socialization is a recognized challenge during this transition, as students must learn how to navigate new settings, internalize the values inherent in clinical learning, and navigate the hidden curriculum of their institutions [19, 23]. Our results are consistent with the work of Prince et al. [19], also adding balance, wellness and career development as themes that students perceive most influence this transition. Our results also show that M3s utilize several helpful strategies such as resilience, leisure activities, healthy habits and efficiency skills to navigate this transition; however, they have the highest reliance upon immature coping mechanisms (e.g. denial, alcohol, suppressing feelings) across the three transitions, pointing to an opportunity for schools to develop coaching programs and interventions centered on resilience and healthy coping strategies. Transitioning to the clinical rotations appears to be a vulnerable period for medical students, one for schools to pay attention to and dedicate resources.

The transition from third-year core clerkships to the fourth and final year of medical school is a challenging period that often receives less attention. This phase is influenced by contrasting factors, including increased schedule flexibility combined with a sense of lack of control over the sequence of rotations, career and specialty choices, navigating residency applications, increased responsibilities of the acting-internships roles, residency match, and graduation [24, 25]. In one survey-based study of fourth-year medical students entering different specialties, students identified preparing for residency as the main purpose of fourth year, while maximizing the likelihood of matching into their residency of choice and gaining a broad educational experience [24]. Our findings align with this prior work, where career development is the most represented factor theme for M4s. On the other hand, our results shed light on what appears to be a distinct theme of factors that influence M4 transition not cited at all by the other classes -- non-medical logistics. This theme encompasses factors like impact of finances, planning away rotation site(s), and scheduling. This highlights the unique demands of preparing and applying to residency, which imposes significant time and financial burdens upon students.

Transitioning to the first year of graduate medical education in residency, learners provide patient care under the supervision of senior residents and faculty. Many have difficulty adjusting to their new roles, responsibilities, and workload of intern year [26]. This challenge is compounded by the variability of fourth-year requirements across medical schools, causing first-year residents to have different clinical competencies and foundations of knowledge [27]. Consequently, interns can experience a high level of stress and burnout [28]. A variety of strategies and skills-based educational interventions have been developed by schools and residency programs to assist students in their transition to residency [12, 14, 27]. Notably, we found the theme of “Knowledge, knowledge application and skills” (e.g. learning hospital logistics and the EMR, placing orders, basic knowledge, etc.) as most impactful for this transition, further supporting the need for such curricular interventions. We also found that interns are developing resilience and preventing burnout by prioritizing work and becoming more efficient, engaging in leisure activities (vacation, spending time with co-residents, reading for pleasure), and developing healthy practices (e.g. exercise, therapy, developing habits) and mature coping mechanisms (e.g. enjoying 4th year in order to start residency mentally refreshed & ready to go). While some of these strategies may be specific to the intern year, looking for ways to support or encourage these in earlier learners may ease prior transitions.

### Strengths and limitations

Our study adds to prior literature on medical education transitions in several ways. First, a stakeholder-oriented approach facilitated collection of qualitative data from the targeted group, enabling evaluation of the status of these transitions from multiple perspectives. This approach ensures that those who can provide the most applicable feedback regarding the strengths and weaknesses of the current system can inform the next generation of medical education curricula. Second, our study collected cross-sectional data from three different years of trainees, providing a unique perspective and a deeper understanding of the similarities and differences inherent to each transition.

Another strength of this study is its ability to map identified transition factors and strategies within established transition frameworks [19, 20]. This study also introduces new themes, such as “Balance and wellness,” “Non-medical logistics,” “Immature coping mechanisms,” and “School/program support and educational tools,” which were not previously identified. Moreover, this work employs pre-existing frameworks to analyze the three transitions in question. The Westerman transition framework [16] initially described the factors characterizing the transition from resident to attending physician, outlining disruptive elements, coping strategies, and personal development and outcomes. Further application of this framework at the pre-clinical level has shown those transition themes are also relevant earlier in medical education [2].

Some limitations of this study should be noted. First, it was conducted at a single institution, limiting the generalizability of results. However, while our findings reflect the curricular structure, context, and institutional culture at UCF COM, these are not significantly different from the majority of M.D. schools in the US as all programs must meet the Liaison Committee on Medical Education (LCME) accreditation standards. Second, our study comprised a limited number of NGT sessions with a relatively small participant pool raising a concern for selection bias. However, considering the exploratory nature of this research and the well-established NGT method guidelines [17, 18], our three groups likely produced a comprehensive list of responses representative of the transitions at our institution. The substantial volume of responses and our familiarity with NGTs suggest that the three meetings held to address our two research questions were likely adequate and that the information gathered from additional sessions would probably not justify the added effort and expense.

In summary, identifying the greatest challenges encountered by students and first-year residents during their transitions can help address areas where additional support is needed to foster smoother transitions. These findings are timely, as undergraduate medical education curricula are currently undergoing changes to address the challenges intrinsic to these student transitions. Future directions can explore how the impact of these factors and strategies change as interventions to the curricula are applied.

## Conclusion

Many medical schools across the US are undergoing competency-based curricular transformations, aiming to shorten the pre-clinical learning period, introduce students to the clinical environment earlier, integrate basic science content to the clinical years, and make the post-clerkship time more flexible and customized to best prepare learners for residency training [28]. Regardless of the curricular structure and the timing of transitioning to clinical rotations, medical educators should address these transitions and offer students a scaffolded structure and resources to succeed. Insights from our findings can enable medical schools to implement evidence-based curricular and/or extracurricular interventions and strategies aimed at improving student well-being and success during transition periods.

### Electronic supplementary material

Below is the link to the electronic supplementary material.

Supplementary Material 1

Supplementary Material 2

Supplementary Material 3

Supplementary Material 4

## Data Availability

Derived data supporting the findings of this study are available within the article and/or its supplementary materials. Any additional dataset used and/or analyzed during the current study available from the corresponding author on reasonable request.

## Abbreviations

NGT: Nominal Group Technique
M3: Third-year medical student
M4: Fourth-year medical student
PGY-1: Post-graduate year 1
GME: Graduate medical education
UCF COM: University of Central Florida College of Medicine
