# Supplementary material for: Understanding learners’ experiences across three major transitions in undergraduate medical education
Source: BMC Med Educ. 2024 Jul 11;24:748. doi: 10.1186/s12909-024-05422-1 (PMC11241916; doi:10.1186/s12909-024-05422-1)
Supplement: Supplementary file 1 — Supplementary Material 1 [file 12909_2024_5422_MOESM1_ESM.docx]

| M3 Students' Ranking Factors Impacting the M2-M3 Transition** | Sum of ranks† | Number of students Selecting the response |
| --- | --- | --- |
| Lack of autonomy over own clerkship hours | 15 | 5 |
| Needing to be constantly enthusiastic and eager | 15 | 4 |
| Shifting from objective to subjective grading | 13 | 3 |
| Working with new classmates outside of usual friend group | 11 | 4 |
| Starting as the individual at the bottom of the hierarchy | 10 | 3 |
| Not knowing roles and responsibilities, largely feeling like a superfluous team member | 10 | 3 |
| Having to study after working long hours at clerkship | 9 | 2 |
| Investing emotionally in my patients | 5 | 1 |
| Managing increased expectations that come with being a third year | 5 | 1 |
| The expectation that you would keep a positive attitude and open mind even in rotations not  interested in | 4 | 1 |
| Navigating different personalities of peers as team members | 4 | 1 |
| Exposure to life and death situations | 4 | 1 |
| Having attendings, residents, clerkship directors seemly unaware that there is a shelf at the end of  the rotation | 4 | 1 |
| Difficult to maintain relationships and find time for friends | 3 | 2 |
| Significantly more clinical interaction and patient care | 3 | 1 |
| Working with real patients, not actors | 3 | 1 |
| Loneliness due to being more isolated from classmates | 3 | 2 |
| Learning to prioritize important things in your life due to less free time | 3 | 1 |
| Everyone starting at level playing field because 3rd year is unknown (clean slate) | 2 | 1 |
| Remembering why I’m in medical school | 2 | 1 |
| Despite finding your favorite specialty having to doing everything except for the rest of the year | 2 | 1 |
| Having school work fall from being the only priority | 1 | 1 |
| Having to navigate multiple new hospitals/EMRs | 1 | 1 |
| Variability of free time in between and within rotations | 1 | 1 |
| Always feeling guilty when not studying | 1 | 1 |
| Huge emphasis on professionalism | 1 | 1 |
| Much closer to match & graduation | 0 | 0 |
| Long hours | 0 | 0 |
| Variability of location | 0 | 0 |
| Trying to impress attendings/ residents | 0 | 0 |
| Always being watched | 0 | 0 |
| Navigating different personalities of superior | 0 | 0 |
| Limited liability & consequences for actions, able to learn | 0 | 0 |
| Evaluating each rotation as a possible career opportunity | 0 | 0 |
| Being book smart but not clinically smart | 0 | 0 |
| Less flexibility in schedule | 0 | 0 |
| How Step clouded your experience (PTSD vs finding out) | 0 | 0 |
| Comparing self to other M3s (peers) | 0 | 0 |
| Being compared to other M3s (peers) | 0 | 0 |
| Surprise of seeing real pathology | 0 | 0 |
| More exhaustion | 0 | 0 |
| Excessive busy work | 0 | 0 |
| Having a space in the hospital to relax, sit, escape (ex: residents lounge) | 0 | 0 |
| Having to become more adaptable | 0 | 0 |
| Accepting that you usually won’t know the answer | 0 | 0 |
| Really long daily drives | 0 | 0 |
| 4th year/upper classmen cluing me in on the fact that shelf is the most important determinate for  your grade | 0 | 0 |
| Lack of preparation to be in a hospital including how to act, roles, ect | 0 | 0 |
| Not taught certain clinical skills | 0 | 0 |
| Interacting with other members of medical team (nurses, PAs, ect) | 0 | 0 |
| Becoming a better learner | 0 | 0 |
| New team every week | 0 | 0 |
| Staying up all night | 0 | 0 |
| Remembering to do clerkship assignments | 0 | 0 |
| Cost effective medicine – labs, tests cost money | 0 | 0 |
| More pressure to decide on specialty | 0 | 0 |
| Taking a break/ vacation between Step and first rotation | 0 | 0 |
| Having a solid knowledge base after step 1 and only needing to supplement for first rotation vs  later in the year | 0 | 0 |
| Sounding more like doctor now | 0 | 0 |
| Actively participating in a code | 0 | 0 |
| More physically demanding | 0 | 0 |
| Resident with a sense of humor who clearly liked teaching who made transition easier | 0 | 0 |
| Mandatory obligations from school with required attendance | 0 | 0 |
| Taking out stress on people close to you | 0 | 0 |
| Commiserating/identifying shared experience with peers | 0 | 0 |
| Crying a lot | 0 | 0 |
| **Based on responses from 9 M3 students  †Calculated by summing the ranks (5 = most important, 4 = fourth, 3 = third, 2 = second and 1 = least important) assigned to the response. The higher the score, the greater the perceived importance | | |

| M3 Students' Ranking of Strategies to Manage the M2-M3 Transition** | Sum of ranks† | Number of students Selecting the response |
| --- | --- | --- |
| Talking to M4s & PGY1s about their experiences, resources to use, and general advice. | 19 | 4 |
| Develop strong sense of self. | 12 | 3 |
| Setting aside time for myself. | 9 | 3 |
| Engaging in mindfulness activities such as yoga, meditation, and going on walks. | 8 | 2 |
| Making time for exercise and fitness throughout the week. | 7 | 2 |
| Talking family frequently. | 7 | 2 |
| Actively learning during each clerkship. | 6 | 2 |
| Learning about the logistic of the clerkship from other M3 students who completed the clerkship. | 5 | 1 |
| Setting expectations with residents and attendings on my team. | 5 | 1 |
| Selecting a few resources to use for each clerkship and spending most of my time with these. | 5 | 2 |
| Talking to family frequently. | 5 | 2 |
| Reddit/ memes | 4 | 1 |
| Stopped trying to constantly impress everyone all the time | 4 | 1 |
| Better time management with more organized to do list | 4 | 1 |
| Optimize morning routine | 4 | 1 |
| Mental health counselling/ therapy (Dr. Daly resources) | 4 | 3 |
| Recognizing & Advocating for the value of your own time | 4 | 1 |
| Joke/ Humor | 3 | 1 |
| Use hospital downtime to study | 3 | 1 |
| Non-medical Hobbies (ex: scuba diving, playing music, reading) | 3 | 1 |
| Stop caring so much | 2 | 1 |
| Define close friends & spend more time with them | 2 | 1 |
| Talking to significant other/ spouse | 2 | 1 |
| Remaining curious/ asking questions | 2 | 1 |
| Strict sleep schedule compared to 2nd year | 2 | 2 |
| Keep study habits constant | 1 | 1 |
| Kept quiet to not draw attention to self about lack of knowledge/ pimp prevention | 1 | 1 |
| Thinking about long term goals not just short term | 1 | 1 |
| Taking time to cook dinner every night | 1 | 1 |
| Set a schedule |  |  |
| Reddit posts/ other forums for medical school advice |  |  |
| Suppress my feelings |  |  |
| Play with/pet cats/ walk dogs |  |  |
| Talking to non-medical school people |  |  |
| Think of things I can do on my down time (driving) ex podcasts |  |  |
| Reading books for pleasure |  |  |
| Venting to friends |  |  |
| Caring less about evaluations |  |  |
| Rant to mom & boyfriend |  |  |
| Using nights/weekends to do activities unrelated to school |  |  |
| Only did what was asked of me, nothing more |  |  |
| Find a specialty advisor |  |  |
| Watch tv, find Netflix show |  |  |
| Relied on denial as a coping mechanism |  |  |
| Eating candy/ baking |  |  |
| Ask residents, attendings on team for feedback about performance |  |  |
| Make excuses to leave |  |  |
| Cleaning/ organizing living space |  |  |
| Take a lot of bathroom breaks |  |  |
| Find out if you can access hospital EMR at home |  |  |
| Eating well |  |  |
| Caffeination |  |  |
| Wine |  |  |
| Taking things one day at a time |  |  |
| Tried study drugs |  |  |
| Preparing for patients & cases the day before |  |  |
| Improving sleep hygiene |  |  |
| Shopping |  |  |
| Having a mantra during difficult times |  |  |
| Not thinking or talking about Step 1 |  |  |
| Making use of smart residents (who like to teach) |  |  |
| Plan a vacation |  |  |
| Self-reflection (ex: journal, write poems) |  |  |
| **Based on responses from 9 M3 students  †Calculated by summing the ranks (5 = most important, 4 = fourth, 3 = third, 2 = second and 1 = least important) assigned to the response. The higher the score, the greater the perceived importance | | |
