# Supplementary material for: Understanding learners’ experiences across three major transitions in undergraduate medical education
Source: BMC Med Educ. 2024 Jul 11;24:748. doi: 10.1186/s12909-024-05422-1 (PMC11241916; doi:10.1186/s12909-024-05422-1)
Supplement: Supplementary file 2 — Supplementary Material 2 [file 12909_2024_5422_MOESM2_ESM.docx]

**List of Appendices**

Appendix 1: List of responses generated in M3 NGT

Appendix 2: List of responses generated in M4 NGT

Appendix 3: List of responses generated in PGY1 NGT
