# Supplementary material for: Understanding learners’ experiences across three major transitions in undergraduate medical education
Source: BMC Med Educ. 2024 Jul 11;24:748. doi: 10.1186/s12909-024-05422-1 (PMC11241916; doi:10.1186/s12909-024-05422-1)
Supplement: Supplementary file 3 — Supplementary Material 3 [file 12909_2024_5422_MOESM3_ESM.docx]

| M4 Students' Ranking of Factors Impacting the M3-M4 Transition | Sum of ranks† | Number of students Selecting the response |
| --- | --- | --- |
| Choosing a specialty | 19 | 4 |
| Changes in career and educational priorities | 15 | 4 |
| Having the freedom and flexibility to make my own schedule | 14 | 4 |
| Going to a new place to do rotation, leaving the state and living in a new place | 13 | 5 |
| More confident in fourth year than third year | 9 | 3 |
| Step 2 CK and CS as a milestone | 8 | 4 |
| More practical knowledge and experience | 6 | 2 |
| My significant other and family | 5 | 1 |
| First day on away rotation | 5 | 1 |
| Different mentality | 5 | 1 |
| Availability of finances | 5 | 2 |
| Able to make those closest to me a priority | 3 | 1 |
| Pre-planning for schedules and rotations, and being ready for it to change | 3 | 1 |
| Learning for myself rather than exams, self-motivated lifelong learning | 3 | 2 |
| Learning about one specific field of medicine | 2 | 1 |
| Not being the lowest on the totem pole | 2 | 1 |
| Being constantly evaluated on daily basis | 1 | 1 |
| Advisors that I trusted | 1 | 1 |
| Maintain work-life balance | 1 | 1 |
| Good roommates | 0 | 0 |
| International experience | 0 | 0 |
| Where my away rotations were scheduled | 0 | 0 |
| New responsibilities | 0 | 0 |
| Passion for chosen specialty | 0 | 0 |
| Making up missed third year rotation | 0 | 0 |
| Developments in my career goals that were always changing | 0 | 0 |
| Openness with friends and family about the fourth year process and struggles | 0 | 0 |
| Better established relationship with peers and mentors | 0 | 0 |
| Relationship with attendings and nurses | 0 | 0 |
| Opportunity to just focus on patient care | 0 | 0 |
| Mental health going into fourth year improved from going into third year | 0 | 0 |
| Counseling and resources | 0 | 0 |
| **Based on responses from 8 M4 students  †Calculated by summing the ranks (5 = most important, 4 = fourth, 3 = third, 2 = second and 1 = least important) assigned to the response. The higher the score, the greater the perceived importance | | |

| M4 Students' Ranking of Strategies to Manage the M3-M4 Transition** | Sum of ranks† | Number of students Selecting the response |
| --- | --- | --- |
| Mentorship – COM alumni/residents | 22 | 5 |
| Mentorship – faculty/specialty advisors | 15 | 4 |
| Sound-boarding with peers | 14 | 4 |
| UCF COM specialty guide | 7 | 2 |
| Working hard throughout rotations | 5 | 1 |
| Checking in regularly with advisors and mentors frequently | 5 | 1 |
| Focusing on patient care over grades | 5 | 1 |
| Therapy sessions with Dr. Daly | 4 | 1 |
| Motivated self-learning | 4 | 1 |
| Shared information/keeping them up to date about timelines with family | 4 | 1 |
| Feeding off my passion for the field | 3 | 1 |
| Specialty specific online resources (ex: EMRA) | 3 | 1 |
| Carefully planning away rotations (location, program, timing, housing, transportation) | 3 | 2 |
| Reframing mistakes as positive learning experiences | 3 | 1 |
| Don’t procrastinate | 3 | 1 |
| Time for self-reflection (ex. prayer) | 3 | 1 |
| Spending time with friends doing non-school stuff | 2 | 1 |
| Charting outcomes in the match | 2 | 1 |
| Becoming efficient at traveling, get TSA precheck | 2 | 1 |
| Don’t be emotional or offended by anything | 2 | 1 |
| Take a break before third and fourth year | 2 | 1 |
| Letting myself relax when I have time off | 2 | 2 |
| Prioritizing my personal interests | 1 | 1 |
| MSPE meeting | 1 | 1 |
| Scheduling in order to dedicate adequate time for Step 2 studying | 1 | 1 |
| Becoming self-aware about positive attributes by talking to parents and significant other | 1 | 1 |
| Being more sociable and engaged on rotations | 1 | 1 |
| Making time to exercise | 0 | 0 |
| Visiting and spending more time with family | 0 | 0 |
| Making sleep a priority | 0 | 0 |
| Planning rotations I actually enjoy | 0 | 0 |
| Try to create good impressions on programs on away rotations | 0 | 0 |
| FREIDA | 0 | 0 |
| Eating healthier foods | 0 | 0 |
| Buying frozen and easy to prep food | 0 | 0 |
| Dog play dates | 0 | 0 |
| Getting along with other rotators | 0 | 0 |
| Going on international trips to refocus and recharge | 0 | 0 |
| Control over own schedule using Oasis | 0 | 0 |
| Netflix | 0 | 0 |
| Limiting duration of time for known stressors, such as step 2 study time | 0 | 0 |
| Roadmap to Residency | 0 | 0 |
| Taking time to find an advisor that I can trust and constantly be in contact with | 0 | 0 |
| Having fun events to look forward to with friends and family | 0 | 0 |
| Develop specific future career goals | 0 | 0 |
| M4 specific GPS | 0 | 0 |
| Stay organized make lots of excel spreadsheets | 0 | 0 |
| Always be available and enthusiastic on rotations | 0 | 0 |
| Residency Director Survey (specialty specific) | 0 | 0 |
| Made and used H&P templates | 0 | 0 |
| Getting back in touch with fulfilling hobbies | 0 | 0 |
| Volunteering | 0 | 0 |
| Oversold my confidence | 0 | 0 |
| Planning who is going to write LORs, knowing program requirements | 0 | 0 |
| Using credit card that redeems flight miles | 0 | 0 |
| Having family help (ex: passwords for email, bills, interview invites) | 0 | 0 |
| Capstone Week, intro to 4th year | 0 | 0 |
| Avoided stressful events alone, did Step 2 CS and CK with other people | 0 | 0 |
| Specialty specific meeting with above class | 0 | 0 |
| Looked at fourth year as an opportunity for new experiences | 0 | 0 |
| Online med ed intern videos | 0 | 0 |
| Practicing interviewing | 0 | 0 |
| Being proactive and helpful on rotations | 0 | 0 |
| Learning to network | 0 | 0 |
| Normalizing anxiety by talking with friends | 0 | 0 |
| Reminding myself there are no exams after Step 2 | 0 | 0 |
| Be able to improvise on interviews and rotations | 0 | 0 |
| Dedicated time to reflect on third year, and what to do differently for fourth year | 0 | 0 |
| Introduce yourself to everyone and be a team player | 0 | 0 |
| Specialty specific reddit spreadsheets | 0 | 0 |
| Getting an annual pass to Universal Studios | 0 | 0 |
| **Based on responses from 8 M4 students  †Calculated by summing the ranks (5 = most important, 4 = fourth, 3 = third, 2 = second and 1 = least important) assigned to the response. The higher the score, the greater the perceived importance | | |
