# Supplementary material for: Understanding learners’ experiences across three major transitions in undergraduate medical education
Source: BMC Med Educ. 2024 Jul 11;24:748. doi: 10.1186/s12909-024-05422-1 (PMC11241916; doi:10.1186/s12909-024-05422-1)
Supplement: Supplementary file 4 — Supplementary Material 4 [file 12909_2024_5422_MOESM4_ESM.docx]

| PGY1 Ranking of Factors Impacting the M4-PGY1 Transition | Sum of ranks† | Number of students Selecting the response |
| --- | --- | --- |
| Hours at work | 14 | 3 |
| Learning the logistics of the how the hospital works | 9 | 2 |
| Tons of responsibility | 7 | 2 |
| Amount of direct patient care | 7 | 3 |
| Learning the new EMR | 5 | 1 |
| Volume of patients seen | 5 | 2 |
| Putting in orders | 4 | 1 |
| Being thrown into the ICU | 4 | 1 |
| Free food | 4 | 2 |
| Rotating through certain fields of medicine I have less interest in | 4 | 1 |
| Lack of sleep | 3 | 1 |
| Amount of knowledge I had | 3 | 1 |
| Workload | 3 | 1 |
| Figuring out new expectations | 2 | 1 |
| Learning what resources were available and how to use | 2 | 2 |
| Time always at a premium | 2 | 1 |
| New attendings | 1 | 1 |
| Stress about aspects of life outside of residency | 0 | 0 |
| Using skills developed from M4 AIs such as clinical judgement | 0 | 0 |
| Finding good mentors who understand my career path | 0 | 0 |
| Interpersonal relationships | 0 | 0 |
| Knowledge gained during M2 | 0 | 0 |
| Realizing how lax M4 was in comparison | 0 | 0 |
| Learning time management skills & organization | 0 | 0 |
| New co-residents | 0 | 0 |
| Morning Report | 0 | 0 |
| Needing to learn on the job- harder to find time for outside studying | 0 | 0 |
| **Based on responses from 5 PGY1 residents  †Calculated by summing the ranks (5 = most important, 4 = fourth, 3 = third, 2 = second and 1 = least important) assigned to the response. The higher the score, the greater the perceived importance | | |

| PGY1 Ranking of M4-PGY1 Transition Strategies | Sum of ranks† | Number of students Selecting the response |
| --- | --- | --- |
| Vacation | 9 | 2 |
| Vent to significant other, family, close friends | 7 | 3 |
| Exercise | 7 | 2 |
| Share the workload with peers | 5 | 1 |
| Spending time in social group activities outside of medicine (friends/family) | 5 | 1 |
| Reading Up-To-Date | 5 | 1 |
| Ensuring to eat 3 square meals a day | 5 | 1 |
| Become more efficient | 5 | 2 |
| Spending time with co-residents | 4 | 1 |
| Get advice from senior residents | 3 | 1 |
| Commiserating with peers | 3 | 1 |
| Enjoying 4th year in order to start residency mentally refreshed & ready to go | 3 | 1 |
| Program very supportive | 3 | 1 |
| Turn off hospital phone when outside of hospital | 3 | 2 |
| Therapy | 3 | 2 |
| Remembering what I'm going into (not medicine) | 2 | 1 |
| Learn Spanish to not rely on translator | 2 | 1 |
| Drawing from experience gained from AI/ICU during 4th year | 1 | 1 |
| Forming habits | 0 | 0 |
| Watch TV | 0 | 0 |
| Reading for future specialty | 0 | 0 |
| Eat ice cream | 0 | 0 |
| Find mentor in future specialty | 0 | 0 |
| Reading for pleasure | 0 | 0 |
| **Based on responses from 5 PGY1 residents  †Calculated by summing the ranks (5 = most important, 4 = fourth, 3 = third, 2 = second and 1 = least important) assigned to the response. The higher the score, the greater the perceived importance | | |
